# Spatially-Correlated Risk in Nature Reserve Site Selection

**DOI:** 10.1371/journal.pone.0146023

**Published:** 2016-01-20

**Authors:** Heidi J. Albers, Gwenlyn M. Busby, Bertrand Hamaide, Amy W. Ando, Stephen Polasky

**Affiliations:** 1 Haub School of Environment and Natural Resources, Department of Economics and Finance, University of Wyoming, Laramie, Wyoming, 82072, United States of America; 2 GreenWood Resources, Portland, Oregon, 97201, United States of America; 3 Department of Economics, Social and Political Sciences, Université Saint-Louis, Brussels, Belgium; 4 Department of Agricultural and Consumer Economics, University of Illinois, Urbana, Illinois, 61801, United States of America; 5 Department of Applied Economics, University of Minnesota, Saint Paul, Minnesota, 55108, United States of America; US Army Engineer Research and Development Center, UNITED STATES

## Abstract

Establishing nature reserves protects species from land cover conversion and the resulting loss of habitat. Even within a reserve, however, many factors such as fires and defoliating insects still threaten habitat and the survival of species. To address the risk to species survival after reserve establishment, reserve networks can be created that allow some redundancy of species coverage to maximize the expected number of species that survive in the presence of threats. In some regions, however, the threats to species within a reserve may be spatially correlated. As examples, fires, diseases, and pest infestations can spread from a starting point and threaten neighboring parcels’ habitats, in addition to damage caused at the initial location. This paper develops a reserve site selection optimization framework that compares the optimal reserve networks in cases where risks do and do not reflect spatial correlation. By exploring the impact of spatially-correlated risk on reserve networks on a stylized landscape and on an Oregon landscape, this analysis demonstrates an appropriate and feasible method for incorporating such post-reserve establishment risks in the reserve site selection literature as an additional tool to be further developed for future conservation planning.

## Introduction

Networks of nature reserves offer protection to biodiversity and sets of species. Because constraints limit the amount of area allocated to biodiversity conservation, the reserve site selection (RSS) and related literatures examine the issue of selecting parcels to be conserved across a landscape with species distributed across that landscape. In the simplest maximal covering problem, the objective is to choose parcels for preservation to “cover” or represent the greatest number of species in the reserve system, with no additional value for protecting a species twice [1,2]. Building from that foundation, this literature solves the maximal species coverage problem for a variety of settings including addressing uncertainty about species occurrence through probabilistic approaches and considering issues of contiguity of reserves through connectivity and border length constraints. The probabilistic RSS models typically address uncertainty about species presence/absence at a particular location in a landscape with an objective of maximizing the expected number of species covered by a reserve network (e.g. [3,4,5]). Because the probability of a species being present on a parcel is less than one, it can be optimal to conserve two or more plots with the same species to increase the expected number of the species conserved.

In many cases, the probability that habitat and its species survive within the reserve network may not be independent across space. As part of the SLOSS (single large or several small) debate [6], many biologists suggest that spatially aggregated, contiguous or connected reserves increase the survival probabilities of many species [7,8,9,10]. With that biological information, many studies within the RSS literature extend the modeling framework to include connectivity or adjacency requirements as tools to create agglomerated reserve networks (e.g. [11,12,13,14] and [15] for a review). [16] consider the impact of land use change, including fragmentation, outside of reserves on species within reserves through neighborhood habitat effects, but consider only the vulnerability of a non-reserve parcel to conversion to urban or agricultural land rather than naturally occurring risks to species within reserves, albeit in a dynamic context. The “several small” side of the SLOSS debate has received less attention, but argues that the risk of disease or other factors killing all or most extant members of a species is greater with one large reserve than with several unconnected reserves. [17] and [18] are two of the few papers exploring constraints on both proximity of reserve parcels and distances between parcels, with both papers using a non-probabilistic setting.

This paper considers uncertainty and risks posed by agglomerated reserves from a different perspective. Concerning uncertainty, we assume that the presence/absence data are perfect but the uncertainty lies in the fact that species in the reserve face risk of destruction from a variety of naturally-occurring sources including fire, defoliating pests, and disease; meaning that species face threats, and are vulnerable to those threats, even when present within the reserve [19]. Hence, we maintain the probabilistic approach of earlier models and maximize the expected number of species in the reserve following random destructive events. When the habitat of a parcel is eliminated due to these destructive forces, the species on those parcels are no longer protected by the reserve system. Converting the standard coverage model to one that maximizes the expected number of species in the reserve network based on the probability of their habitat’s survival leads to similar redundancies in species coverage to those found in the probabilistic presence/absence data case.

This modeling effort is a step toward informing the “several small” side of the SLOSS debate by considering spatially correlated risks during reserve design decisions. Indeed, because many diseases, pests, invasive species, and fires that cause habitat destruction spread spatially from a starting point, they pose risks to parcels that are connected to each other, as are parcels within a “single large” reserve network. Although the risk associated with the starting point, such as the location of the lightning strike that ignites a fire, may be independent across a landscape, once that threat to habitat initiates, the risk it poses on the landscape is spatially-correlated to the initial location. With spatially-correlated risk, the probability of species survival on any one parcel is no longer independent of the risks facing neighboring parcels.

The issue of spatially-correlated risk is important and relevant to conservation planning. For example, wildland fires begin at the point of a lightning strike and, in a homogeneous landscape, spread radially from that point, damaging potentially large areas of contiguous habitat. In the last decade, western U.S. forests have seen an increase in large, and often stand-replacing, fires such as Oregon’s 557,628 acre Long Draw fire in 2012. Habitat-damaging pests spread across space from a point of introduction, killing off other species and disrupting habitat in its path. As another example, once introduced in a location, gypsy moths spread slowly from that position and destroy habitat by defoliating trees. Similarly, Mountain Pine Beetle outbreaks damaged over 450,000 acres of forest habitat in 2009 and 2010 [20]. Diseases that kill plant species can also spread from an initial introduction point to disrupt habitat function in large areas. Sudden Oak Death, caused by the pathogen *Phytopthora ramorum*, spreads from host to host within a forest, in addition to other spread mechanisms, and has damaged habitat containing oak trees throughout much of California and southwestern Oregon. Also a radially spreading disease, evidence of habitat damage from Swiss Needle Cast occurred on more than 300,000 acres in Oregon in 2010 [20]. Such fires, pests, and diseases spread through ecosystems and thereby generate spatially- correlated risk to habitat even if the habitat is within a reserve network. Some researchers [21,22,23] incorporate spatially-correlated risks into a metapopulation modeling framework and find that spatially aggregated habitat does not improve species survival and, in fact, may even increase the risk of extinction. Here, we focus on spatially correlated risks to habitat and leave the study of other risks, such as wildlife diseases that spread through contact between individuals, to further study. By focusing on the risks associated with agglomerated reserves, this paper takes a step toward developing systematic conservation planning frameworks that explore the *tradeoffs* between the risks and benefits of configurations of reserves that the RSS literature addresses largely through proximity and separation constraints ([17] and [18]).

In this paper, we develop and solve an optimization model for the expected maximum species coverage problem for spatially-independent and spatially-correlated risk on a stylized landscape; examine how the results of that model address secondary risk management goals; develop a solution method for a larger landscape; and apply that method to determine the best reserve network for a subset of Oregon, subject to simplifying assumptions including those describing species behavior in response to hazards.

The remainder of the paper is organized as follows. The next Section is devoted to modeling reserve site selection with spatially-correlated risk. Sections 3 and 4 apply the models, respectively, to a stylized landscape and to a portion of the State of Oregon. Finally, Section 5 is devoted to discussion and conclusion.

## Modeling Reserve Site Selection with Spatially-Correlated Risk

Following the general description of the probabilistic maximal coverage problem (or expected coverage problem) from [24], we develop a model to choose reserve sites to maximize the expected number of species that survive in the reserve network following a period of stochastic habitat disturbance. Here, each parcel faces a probability that some hazard, such as fire, disease, or pests, will destroy that parcel’s habitat. For simplicity and in keeping with the RSS literature, we assume that the species on non-reserve parcels do not survive, that the destruction of habitat implies that no species survive on that parcel, and that all species present on undisturbed reserve parcels survive. (Although out of the scope of this paper, incorporating spatial species population models into a framework with spatially-correlated risk could capture the characteristics of species’ metapopulations including interactions between within-reserve and non-reserve locations in response to hazards or threats to habitat). The expected number of species that survive in the reserve following a period of stochastic “one-event” habitat disturbance is a function of the distribution of species in the parcels selected for the reserve and the probability that each reserve parcel’s habitat remains after a disturbance. In this section, we develop a modeling framework that considers spatially-independent and spatially-correlated risk in the choice of parcels to conserve in a reserve network. Although many types of hazards are spatially-correlated, for ease of exposition in what follows, we develop the examples with a risk of fire and the resulting burn patterns (Section 2.1), then we develop the model (Section 2.2) that we apply to a stylized landscape and an Oregon landscape (Sections 3 and 4).

### Risk and Burn Patterns

To compare the impact of spatial versus non-spatial risk on reserve design on both a stylized and Oregon landscape, we find the optimal reserve design for a scenario of fires that spread and for one in which fires do not spread. To compare these reserve designs and focus on the role of spatial correlation in risk, we keep the landscape-level risk equal across the scenarios. We define a set, ***B***^**k**^, of all possible patterns of fire disturbance for the fire-spread scenario (*k = 1*) and the no-spread scenario (*k = 2*). We determine the probability of each “burn pattern” within each set of patterns for both scenarios.

Although the probability of ignition itself is independent across space, in the spatially-correlated risk scenario, the fire spreads beyond the ignited parcel into each of the neighboring contiguous parcels. For simplicity, we model spread only to closest neighbors, or first-order, queen contiguity [25]. (The model could be adapted to include other spatial spread patterns based on the characteristics of the landscape and the disturbance.) This spatial spread links the probability of species survival across space. The set of burn patterns ***B***^***1***^ includes the parcels burned from each possible, and equally probable, fire ignition point, including ignition points on all parcels in the focal landscape parcels and on neighboring parcels from which fire spreads into the focal landscape.

The set of burn patterns for spatially-independent fire, ***B***^***2***^, includes the combinations of burned parcels that result from ignitions in the landscape without fire spread. To permit the direct comparison of fire spread case to no fire spread case rather than focusing on the impact of total risk, we insure that the same amount of the landscape burns in the spatial spread and non-spatial fire cases. (Another method of analysis would compare the reserve networks from two different fire scenarios, one with spreading fires and one with one-location fires. In that case, the landscape risk differs dramatically across the two cases, which makes interpreting the impact of the spatially-correlated risk as opposed to the total level of risk level difficult to discern.) Keeping the amount of landscape burned constant across the scenarios implies multiple ignition points to create each burn pattern for this spatially-independent risk scenario. With non-spatial fire risk, the burned parcels are randomly distributed while the spatially-correlated fire risk generates burn patterns with clustered burned parcels.

### The Model

Suppose that a given landscape contains *J* parcels indexed *j* = (1,2,…,*J*) and *I* species *i* = (1,2,…,*I*). On each parcel *j*, the presence (or absence) of each species is known with certainty. Based on ignition probabilities and fire spread characteristics, *B*^*k*^ indexed *b*^*k*^ = {1,2,…,*B*^*k*^} burn patterns can occur during the fire period, each occurring with a known probability *p*_*b*_^*k*^. Because species on parcels outside of the reserve and on burned parcels do not survive, overlaying each burn pattern on the reserve network, given the distribution of species, determines the total number of species surviving in the network and landscape for that burn pattern. Again, this analysis examines only a single burn event and a simplified species response to that event.

Note that many mobile species will flee a burning area, or an area facing defoliation or another habitat hazard, in search of appropriate habitat. That point argues for spatially-explicit wildlife modeling in conjunction with reserve site selection in a dynamic setting, which is beyond the scope of this paper. The ability of wildlife to move from a hazard-location to appropriate habitat would depend on the size of management units, the pace of the habitat destruction, and the mobility of the species but spatially-correlated or spreading risks would likel prove more disruptive to that movement than smaller-scale or non-spreading hazards

In the probabilistic maximal covering problem, the objective is to choose a set of parcels to protect, *x*, to maximize the expected number of species surviving in the reserve network, subject to fire risk. To calculate the expected number of species surviving for a specific reserve network when risk is spatially-correlated (*k* = 1), we sum—over all *B*^*1*^ possible burn patterns in the set ***B***^***1***^—the product of the probability *p*_*b*_^1^ of burn pattern *b*^*1*^ and the number of species that survive in the reserve network given that burn pattern. Similarly, when risk is spatially-independent (*k* = 2), to calculate the expected number of species surviving for a specific reserve network, we sum, over all possible burn patterns in the set ***B***^***2***^, the product of the probability *p*_*b*_^2^ of burn pattern *b*^*2*^ and the number of species that survive in the reserve network after that burn pattern.

The problem can then be formulated as follows:

$$\begin{array}{c} Max\\ x \end{array}\sum_{b^{k}\in B^{k}}\left(p_{b^{k}}\sum_{i\in I}y_{ib^{k}}\right)$$ (1)

Subject to

$$\sum_{j\in J}x_{j}\leq m$$ (2)

$$y_{ib^{k}}=\left\{\begin{array}{c} 1\;if\;(\sum_{j\in N_{ib^{k}}}x_{j})\geq 1\\ 0\;\text{otherwise}\; \end{array}\right\}\;\forall i\in I\;and\;\forall \;b^{k}\in B^{k}$$ (3)

Where

*i*,*I* = index and set of species

*j*,*J* = index and set of parcels

*b*^*k*^,***B***^***k***^ = index and set of burn patterns;

*k* = 1 when risk is spatially-correlated;

*k* = 2 when risk is spatially-independent

*p*_*b*_^*k*^ = probability of burn pattern *b*^*k*^

*m* = upper limit on number of parcels selected for reserve

*N*_*ib*_^*k*^ = set of parcels that contain species *i* after burn *b*^*k*^

*x*_*j*_ = 0,1; 1 if parcel j is selected for reserve, 0 otherwise

*y*_*ib*_^*k*^ = 0,1; 1 if species *i* is present in reserve after burn *b*^*k*^, 0 otherwise

The problem set forth in Eq (1) maximizes the expected number of species present in the reserve network after a period of fire by choosing a set of parcels (***x***) to include in the reserve, subject to a maximum parcel constraint. Constraint Eq (2) sets *m* as the maximum number of parcels that may be selected for reserve and constraint Eq (3) enforces the condition that a species is represented only if a site containing that species after a period of fire was selected for the reserve.

## Application on a Stylized Landscape

**Fig 1:**
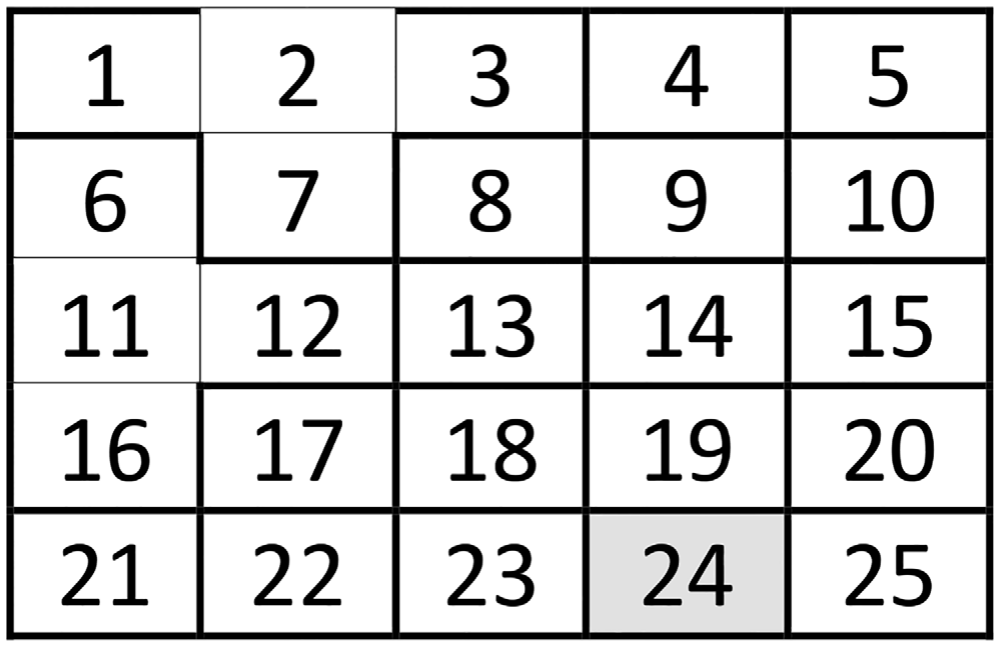
Five-by-five grid stylized landscape.

We first solve the model for a stylized focal landscape consisting of a 5-by-5 grid of parcels (*J* = 25) (Fig 1 shows the focal landscape). Considering its size, the stylized landscape cannot be viewed as a surrogate for a real-world case; its size is small enough to easily calculate all possible combinations of burn patterns and reserve designs—while allowing for a range of species distributions such as hotspots, dispersion and adjacency—but not so large as to be computationally difficult to manage.

Although no management activities take place beyond that focal landscape, 24 neighboring parcels extend the grid by one parcel in all directions around the focal landscape. In the case of spatially-correlated risk through fire spread, ignitions occur with equal probability in each of the 25 focal landscape parcels and in each of the 24 parcels surrounding the focal landscape, from which the fire can spread to adjacent parcels within the focal landscape. This homogeneous distribution of a single ignition event generates 49 possible burn patterns (*B*^*1*^). Burn patterns range in size from 1 parcel, which occurs when the ignition point is in a corner of the broader landscape and spreads to a corner parcel of the focal landscape, to 9 parcels, which occurs when the ignition point’s location is located in the interior 3-by-3 grid of the focal landscape. Each of these burn patterns occurs with the same probability and results in an expected number of burned parcels of 4.6. For the case without fire spread and with spatially-independent risk, the number of burn patterns (*B*^*2*^) is over 2 million. That rounded number is found as such: i) we first compute the probability that 1,2,3,4,6 or 9 parcels burn in each of the 49 spatial burn patterns (no burn patterns with 7 or 8 parcels are possible on a 5-by-5 landscape with fire spreading through first order queen connectivity; burn patterns with 3 parcels burned follow from an ignition point in any of the center 3 parcels just outside of the focal landscape on each of the 4 sides of the landscape) ii) we then sum all possible 1,2,3,4,6, and 9 burn patterns on the 5-by-5 grid of parcels to arrive at 2,235,350. Weighting each number of parcels burned by the probability that that number burns in the fire spread case ensures that the expected number of burned parcels is equal to 4.6, facilitates comparison between the fire spread and no-spread cases, and focuses the comparison on the spatial pattern of the hazard while keeping the total risk to the landscape equal.

A larger landscape would imply more burn patterns with 9 parcels burned because more fires both ignite and spread within the landscape rather than spreading into the landscape from ignition points outside of the focal landscape. The increase in landscape size would also imply that the no-correlation burn pattern would have more parcels within the landscape and a higher expected number of burned parcels—equal to that of the correlated case—due to the relative decline in the importance of ignition points outside the focal landscape as the landscape gets larger.

To explore the impact of spatially-correlated risk on optimal reserve site selection, we examine a range of initial known species distributions generated through Monte Carlo simulations. For each randomly generated species distribution, we identify the optimal reserve design for the case where fires spread from a single ignition point and where fire ignitions are randomly distributed. In the stylized landscape setting, two individual species (*i* = 1,2) are each present on 5 parcels or 20% of total area. Within the 5x5 grid, each parcel contains zero species, a single species, or two species, which we refer to as a “hotspot.” Monte Carlo simulations randomly generate one hundred species distributions with species 1 on five randomly selected parcels and species 2 on five randomly selected parcels.

For each of these 100 species distributions, we solve for the two-parcel reserve (*m* = 2) that maximizes the expected number of species in the reserve following a period of fire disturbance for both the spatially-correlated and spatially-independent fire risk cases. The optimal solution found is specific to the known patterns of species distribution. As with other RSS models, solving this optimal reserve site selection model requires a numerical approach to fully depict the resulting landscapes.

**Fig 2:**
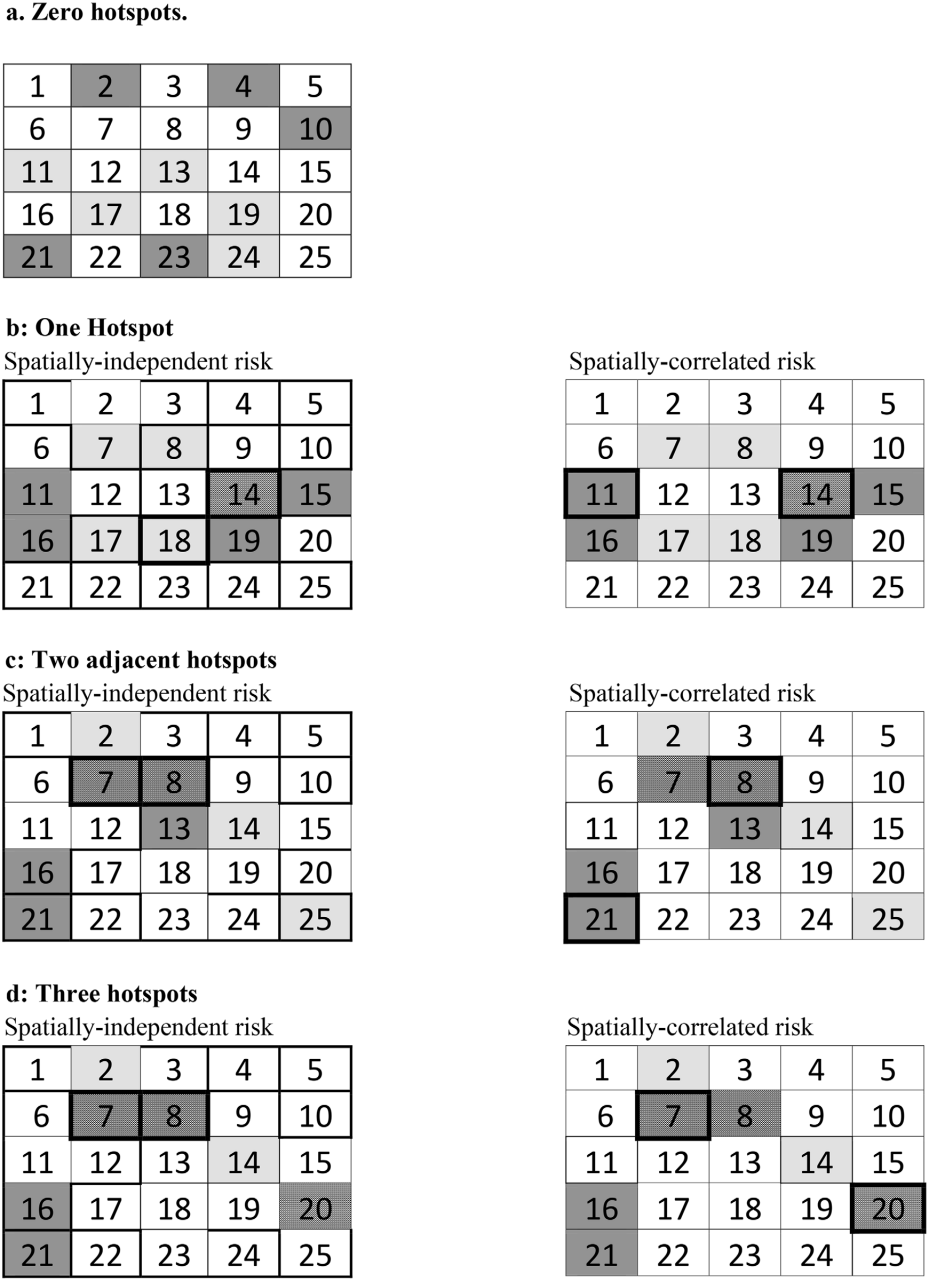
Examples of an optimal reserve design on the stylized landscape. Dark gray = species 1 present; light gray = species 2 present; black and white = biodiversity hotspot; reserve sites marked by bolded parcel outline. (a) Zero hotspot. (b) One hotspot. (c) Two adjacent hotspots. (d) Three hotspots.

We develop a mathematical program to solve the combinatorial optimization problem described in Eqs (1) through (3) and apply that full-optimization model to the stylized landscape. The optimization generates multiple optimal solutions for a given species distribution. Because results depend on the characteristics of the species distribution, we characterize and group the optimal reserve designs according to the number and arrangement of biodiversity hotspots and illustrate examples of optimal reserve designs (Fig 2).

Rather than relying on constraints to prohibit proximity of reserve parcels, this analysis allows for tradeoffs between particularly desirable parcels due to the species located there and the risk to those parcels. Here, the distribution of species interacts with the risk setting to determine the parcels selected for the reserve.

For a species distribution without hotspots, location does not matter in the selection of the optimal reserve design based on maximizing the expected number of species surviving in the reserve (Table 1 and Fig 2a). Even in the spatially-correlated risk case, the expected number of species surviving in the network is the same regardless of whether reserve parcels are adjacent or as separated as possible whenever each species is present in one reserve parcel. However, the reserve’s configuration does affect the probability of particularly good or bad conservation outcomes that might be secondary considerations of reserve managers. Despite the spatial spread of fire, a pattern with adjacent reserve parcels creates the largest probability that both species survive, which occurs when neither parcel in the reserve burns. This result derives from the low number of burn patterns that affect both clustered parcels in the reserve. When reserve parcels are further from each other, more burn patterns affect at least one reserve parcel, making it less likely that both species will survive fire. Looking at the chance of bad conservation outcomes, however, demonstrates that reserve systems with adjacent parcels also create the largest probability that both parcels’ habitats burn and that no species survive. A single large fire can eliminate both species in the reserve network when reserve parcels are separated by one parcel, but the probability of this burn pattern occurring is small. When reserve parcels are separated by more than one parcel, however, a single large fire cannot eliminate both species in the reserve, which minimizes the probability of zero species surviving. Although maximizing the expected number of species leads to indifference between agglomerated and disaggregated reserve networks even in the spatially-correlated risk scenario, a more risk-sensitive secondary objective that minimizes the chance of losing half or all species produces a spatially disaggregated reserve network.

**Table 1:** Spatially-correlated Risk and Zero Hotspots.

	Reserve configuration		
	Adjacent parcels	Parcels separated by one	Parcels separated by more than one
Probability **zero** species survive fire	6/49 (0.122)	3/49 (0.061)	0/49 (0.000)
Probability only **one** species survives fire	6/49 (0.122)	12/49 (0.245)	18/49 (0.367)
Probability **both** species survive fire	37/49 (0.756)	34/49 (0.694)	31/49 (0.633)
**Expected Number of Species**	**1.632**	**1.632**	**1.632**

In landscapes with one or more biodiversity hotspots, reserve site locations do generate differences between the two risk scenarios in terms of the optimal reserve network and the expected species protection achieved. In landscapes with one hotspot, the optimal reserve network for the spatially-independent risk case contains the hotspot and any other parcel with one species but the optimal reserve network for the spatially-correlated risk scenario contains the hotspot and a one-species parcel that is located at a distance from the hotspot (Fig 2b). Hence, for species distributions with one or more biodiversity hotspots, decisions about optimal reserve design that ignore the relative location of reserve parcels result in inefficient or suboptimal choices of reserve parcel locations and protect fewer species.

In landscapes with two hotspots (parcels with both species), the optimal reserve network for the spatially-independent risk case contains both hotspots regardless of their location on the landscape (Fig 2c). In the spatially-correlated risk and fire spread case, however, the optimal reserve network only contains both hotspots if they are located at a "safe" distance from each other (the example is not shown graphically here but safety implies that no one spreading fire can burn both parcels). If the hotspots are adjacent, the optimal reserve in the spatially-correlated risk case contains one hotspot and another distant parcel with only one species (Fig 2c). The adjacency of the hotspot creates a high enough probability of fire spreading across both parcels that the expected number of species is higher without both hotspots in the reserve network. The spatially-correlated risk of hazard—here, fire spread—creates an incentive to put distance between reserve parcels, even if that network contains fewer pre-hazard species.

Finally, when three hotspots are present (Fig 2d), the optimal reserve network for the spatially correlated case is located in sites 7 and 20—that is, the model selects the two non-adjacent hotspots while the optimal reserve assuming spatially independent risk contains any two of the three hotspots (either (7,20), (7,8) or (8,20)). If the non-spatial manager selects (7,8) or (8,20) but risks are actually spatially correlated, the species protection provided by the reserve differs from the manager’s expectation (Table 2). The expected number of species from the optimal reserve design derived from recognizing spatially correlated risk is up to 14% greaterthan from the reserve design a non-spatial manager might choose (increasing from 1.76 to 2). In general, we see the greatest difference in the expected number of species when the spatial solution protects all species with certainty (in at least one location) and the non-spatial manager chooses a reserve design where there is a positive probability that zero species will survive. In other words, in such a case, it is critical to consider spatially correlated risk when setting up optimal reserve networks.

**Table 2:** Reserve design with three hotspots facing spatially correlated risk.

Reserve Design	Expected Number of Species	Probability both species survive	Probability zero species survive
(7,20)^a^	2	1	0
(7,8)	1.755102	0.877551	.122449
(8,20)	1.959184	.979592	.020408

^a^: Optimal reserve design with spatially correlated risk and one of three optimal reserve designs with spatially-independent risk.

## Application to an Oregon Landscape

To apply the model to an Oregon landscape, which is a much larger problem, we use a heuristic approach. On the stylized landscape, it is possible to numerically solve the optimal reserve site selection problem with spatially-correlated risk by looking at all possible reserve networks. The computational difficulty of finding an optimal solution increases exponentially with the number of parcels and solving a similar problem for a 289 parcel Oregon landscape is not feasible. Heuristics are often used to solve large problems that cannot be solved by traditional mathematical programming techniques. In particular, in the field of natural resource management, heuristics have been applied to many spatial forest planning and harvest scheduling problems (e.g. [26,27]) and reserve site selection problems (e.g. [28,29,30,31]). We use a simulated annealing algorithm as it has been found to perform well relative to other heuristics, such as genetic and tabu search algorithms [32,33]. However, because it is a heuristic, it does not guarantee the optimal solution but, by thoroughly searching the solution space, we can be certain of a “good” solution.

The Oregon landscape used here is partitioned into 289 hexagon-shaped parcels, each approximately 157,000 acres (635 km^2^). This relatively coarse scale is consistent with the scale of disturbances in Oregon such as fires that can reach nearly 500,000 acres in size (e.g., the 2002 Biscuit Fire), diseases such as Swiss Needle Cast that damaged 300,000 acres in 2009 [34], and pests such as Mountain Pine Beetle that harmed over 450,000 acres in 2009 and 2010 [20]. The size of the parcels in this Oregon example is large enough to represent multiple owners and to make reserve creation over the entire parcel difficult. Even at this scale, as in the many other articles that use this Oregon landscape in an RSS setting, this analysis can be viewed as determining the priority parts of the landscape to target with a range of conservation activities, such as public reserves and private conservation through easements or agglomeration bonuses [35].

The complete or partial data set for Oregon has been widely used in the reserve site selection literature, which facilitated our application of the framework [5,36,37,38]. For each parcel, we have species presence-absence data for 424 terrestrial vertebrate species (S1 Dataset). The average number of species on a single parcel is 204.11 and the minimum and maximum number of species are 165 and 264, respectively. Because the range of species present on each parcel is not extreme, the landscape contains no clear “hotspots” like those in the stylized landscape example. Based on the stylized landscape results, this lack of hotspots in the Oregon dataset suggests that little difference will arise between spatially-correlated risk and non-spreading risk scenarios but the application allows us to demonstrate the ease of incorporating such spatial risk characteristics and to examine differences in the reserve networks’ configurations.

The problem is to choose the optimal reserve containing at most 30 parcels (*m*≤ 30)—approximately 10% of total area, which may be considered low given that the “Aichi Targets” suggest protecting at least 15 percent of terrestrial and inland water areas [39]—to maximize the expected number of species surviving a period of disturbance, here fire. Again, we assume that fire eliminates all species on burned parcels. Because the problem’s scale makes identifying each possible burn pattern computationally difficult, we conduct simulations to generate a range of burn patterns. For the spatially-correlated fire risk case, we conduct 500 fire simulations with each simulated fire beginning from one randomly selected ignition parcel location, chosen from a uniform distribution, and spreading to the 6 adjacent parcels, resulting in 500 burn patterns in the set ***B***^***1***^. That arbitrary number is chosen because it is much greater than the number of parcels on the Oregon landscape (289 parcels) and because it enables computation time to remain reasonable. Increasing the number of simulations above 500 dramatically increased the model’s runtime, perhaps due to parameters of the simulated annealing search algorithm.

To maintain an appropriate risk-equal comparison, for each of the 500 simulations in the fire spread case, we randomly create a burn pattern for a corresponding spatially-independent risk simulation that contains the same number of parcels burned as in the fire spread case with all parcels having equal probability of burning. That process creates 500 burn patterns from the fire igniting in multiple randomly selected parcels to form the burn pattern set ***B***^***2***^. The fire simulations are embedded in the simulated annealing algorithm where they generate the probability of parcels and species surviving a period of disturbance for each reserve network considered.

Our simulated annealing algorithm (a 7-step procedure) is outlined in Table 3. The initial temperature is set at 10 and the annealing schedule, or cooling rate, is 0.995. The computer run time required for a single run of the algorithm is under 30 minutes for the Matlab program run on an Intel Core2 Duo CP processor with 2.49 GHz and 3.5 GB of RAM. The basic structure of the algorithm is to start with a 30-parcel reserve network, simulate fire on the landscape for that reserve network, calculate the expected number of species surviving disturbance, and compare the result to other reserve networks until we find a good reserve with a high number of species conserved; that is, a “good” solution.

**Table 3:** Simulated annealing reserve site selection algorithm.

Step 1	Define an initial thirty-hexagon reserve.
Step 2	Apply fire to landscape and allow it to burn through 500 repeated randomized experiments. Calculate the average number of species remaining in thirty-hexagon reserve after fire simulations.
Step 3	Randomly select a hexagon to leave reserve.
Step 4	Randomly select a hexagon to include in the reserve.
Step 5	Apply fire to landscape and allow it to burn through repeated randomized experiments. Calculate the average number of species remaining in thirty-hexagon reserve after fire.
Step 6	If solution is better than best so far, save as best and current. If not, calculate Boltzman constant—there is some positive probability of accepting a non-improving addition to the thirty-hexagon reserve.
Step 7	Increase counter and return to Step 3.

**Fig 3:**
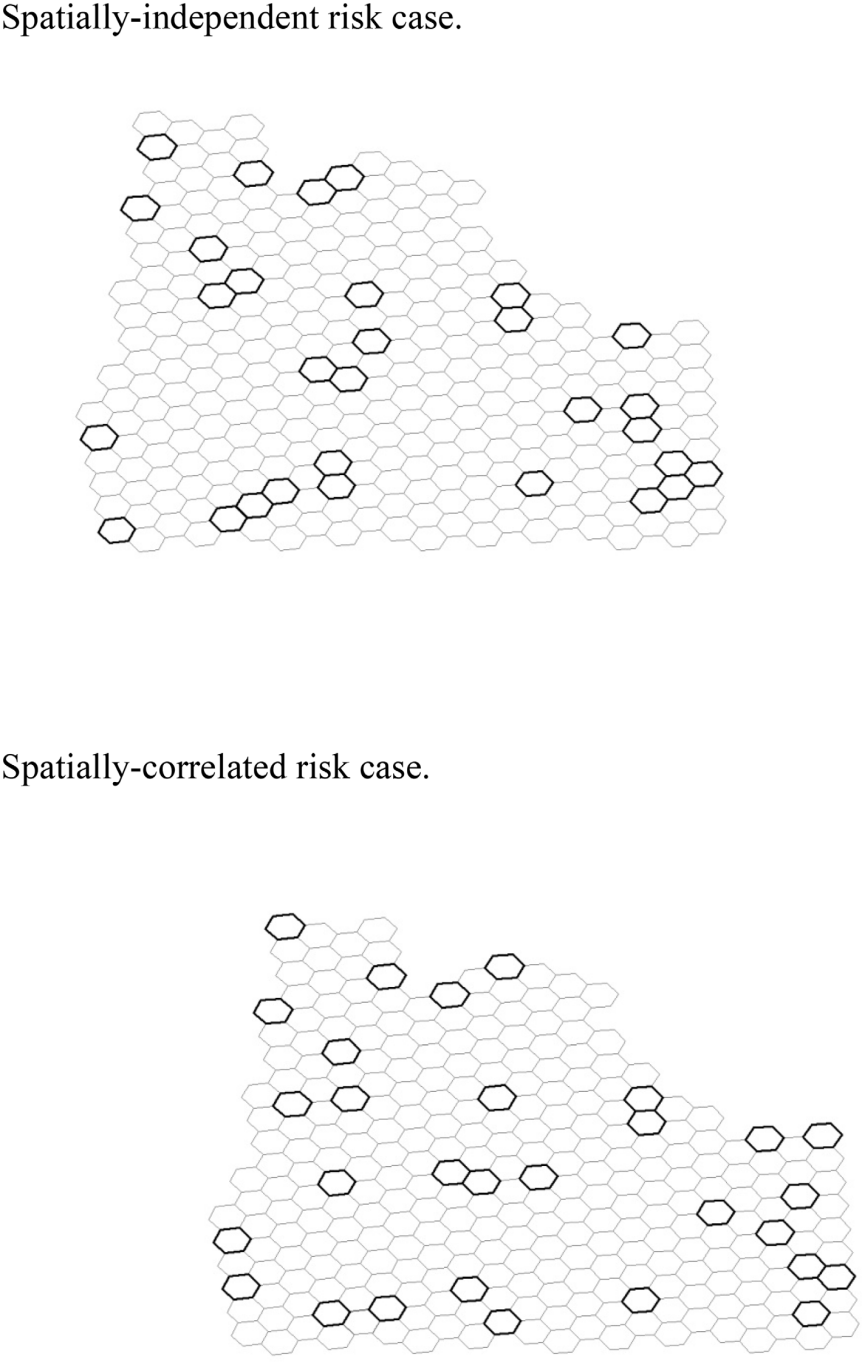
Near-optimal reserve design for the Oregon landscape.

The solutions obtained with the heuristics for both the spatially-independent and spatially-correlated risk cases are illustrated in Fig 3.

The Oregon landscape’s species distribution interacts with the risk setting to determine the optimal reserve network, producing reserve networks with adjacent parcels in both spatially-independent and spatially-correlated risk cases. With spatially-independent risk, the optimal reserve includes 12 shared borders—five pairs of adjacent parcels, one group of three adjacent parcels, and one group of four clustered parcels. With spatially-correlated risk, however, the optimal reserve contains only three shared borders—three pairs of adjacent parcels. Counting the shared borders or adjacencies in the reserve network provides only one measure of the degree of dispersion of the reserve. Because one fire can spread through first-order queen connectivity to include non-adjacent parcels, we develop another measure of the dispersion of the parcels within the reserve networks by counting the number of ignition locations for a spreading fire that burn more than one parcel in the reserve. For example, two reserve parcels that are not adjacent would both burn if the single parcel between them was an ignition point for a spreading fire. This measure of the network’s dispersion incorporates more information about the spatial distribution of reserve parcels in response to spreading fires. The spatially-independent risk reserve case’s optimal network contains 46 such locations while the spatially-correlated risk case’s optimal reserve contains only 28 such ignition points, with ignition points in one of the adjacent parcels or in a parcel adjacent to two reserve parcels. The spatially-correlated risk case generates a more dispersed network as measured by both the number of adjacencies and the number of spreading fires that burn more than one reserve parcel. In the presence of spatially-correlated risk, the benefit from reduced hazard risk makes the dispersed reserve pattern the preferred choice. As in the stylized landscape, however, the species distribution dominates the risk-based aspect of the decision in some cases to produce some reserve locations in close proximity to each other even in the spatially-correlated risk case.

As expected from analyzing the stylized landscape, although the two risk scenarios produce different patterns of reserve networks, they do not differ much in the number of species protected across the risk settings. The best reserve network for the spatially-independent risk case contains an average number of species on a parcel of 211.9, with the minimum and maximum number of species at 182 and 264, respectively. The expected number of species for this reserve network following a period of disturbance without fire spread is 413.15. The chosen reserve network for the spatially-correlated risk case contains an average number of species per parcel of 210.23 and a minimum and maximum number of species of 175 and 264, respectively. The average number of species per parcel in the best reserve is slightly lower than when risk is spatially independent (211.9 versus 210.23), which indicates the negative impact of the spatially-correlated risk on the ability to conserve species in the reserve. The expected number of species for this reserve network is 413.30, slightly greater than in the case with spatially-independent risk.

If the reserve site selection managers ignore the spatially-correlated risk and impose the spatially-independent risk case’s reserve network on a landscape that actually faces spreading fires, they protect fewer species than if they recognized the spatial aspects of risk in establishing the reserve network. This reduced species protection is small in the Oregon example because the landscape contains many parcels with a large number of species [40] applies our framework and methods to data at a smaller scale and finds qualitatively similar results to those of the stylized landscape. However, in other landscapes with fewer species, as shown in the stylized example, the failure to account for spatially-correlated risk could be more costly. Similarly, although fire or other ecological disturbances that spread beyond nearest neighbors would be unrealistically large with the parcel sizes used here, at other scales, more extensive spread of disturbance could induce more distance between reserve parcels and larger losses associated with a failure to consider the spatial dimension of risk to habitat.

Although this Oregon application of our RSS approach does not generate large differences in the expected species protected, the Oregon results demonstrate that this approach can be applied in a real world setting and confirm the essential trade-offs explored on the stylized landscape. The reserve network for the spatially-correlated risk case contains more dispersed parcels than that of the spatially-independent risk case, as expected and as found in the stylized landscape. That dispersed network does not result from an assumption of the model nor a constraint, as in some constraint-focused RSS models, but instead derives from tradeoffs between the spatial characteristics of risk and the distribution and location of species. For example, the reserve network with spatially-correlated risk incorporates three pairs of adjacent reserve parcels because those parcels contain three of the five rarest species in Oregon, each present on only one parcel in the entire landscape. Those parcels could not be included in a reserve network generated with constraints on minimum distances between parcels to protect species from hazards that spread [17,18].

## Discussion and Concluding Remarks

We develop a reserve site selection framework that considers risks to habitat and species within a reserve network when making reserve site selection decisions, and that recognizes that those risks can be correlated across space. We focus on an example of fires that spread from an ignition point, but outbreaks of disease, pest infestations, and invasive species also produce risks to habitat that are correlated across space.

Avenues for future research include extensions to describe other types of spread hazards and other biological reactions to hazards. First, exploration of other types of spatially-correlated risk, such as wildlife disease whose spread depends on individual animals coming into contact with species on adjacent parcels [41], or patterns of the hazard’s spread across space beyond first-order queen, could provide insight for some settings. Similarly, considering other species characteristics and responses such as species movements in response to hazards, species migration, home range needs, and co-location of species would add important biological dimensions to reserve design. For example, with our Oregon landscape’s large parcel size, home range is less critical but edge effects may remain. Also, relaxing the typical RSS assumption that species do not survive outside of the reserve and, here, that the hazard destroys habitat and its species and, instead, modeling the spreading hazard’s partial impact on species in a probabilistic manner could prove a useful departure from the classic RSS literature. In addition, although the examples here consider a known distribution of species, the probabilistic modeling structure permits similar analysis on landscapes with a probability distribution describing species locations.

Given the permanence of many reserve creation decisions, incorporating this spatially-correlated risk perspective into dynamic models of reserve site selection with spatial species population dynamics could improve those long-term decisions, especially in cases in which threats to species outside the reserve include land use conversion and threats to species outside and inside the reserve include neighborhood effects and naturally-occurring hazards [16]. Similarly, broadening the framework away from the single hazard event to examine the spatial-dynamic pathway of hazards, which could include clustering of trees as in the self-organized criticality literature (e.g. [42]) or forest regeneration following repeated fires, would involve linking species persistence probabilities to habitats that change over time in response to hazards and growth. Such an extension would include examining how both hazard risks and habitat suitability for species change over time and address temporally-correlated risks along with spatially-correlated risks. For example, some patterns of reserve parcels might encourage species repopulation of once-burned parcels, with that spatial characteristic interacting with the dynamics of the probability of future hazards.

In addition, the existing model could readily incorporate the probability of species survival as a function of reserve connectivity to demonstrate the tradeoffs between connectivity and dispersed locations in a reserve network. Such an extension of the model would reflect the ecological literature’s emphasis on the benefits of agglomerated reserves and could be applied to determine when and where agglomerated versus dispersed reserves—single large or several small—prove superior in protecting species. That framework would expand both modeling and applications of RSS and systematic conservation planning frameworks because the current proximity and distance constraint-based single-objective RSS models cannot readily examine the tradeoffs between these patterns of reserve networks.

In contrast to the RSS literature’s emphasis on benefits from agglomerated reserves, our analysis explores the possible costs of connectivity that derive from spatially-correlated risk, which emphasizes the benefits of “several small” or more dispersed reserve networks from that literature. Which factor—benefits from agglomeration versus risks to connectivity—dominates will depend on the particular setting. The stylized and Oregon examples illustrate that including spatially-correlated risk in a theoretical and real-world setting can influence optimal reserve design. Yet, even without direct benefits from connectivity in the reserve network and with spatially-correlated risk, the best Oregon reserve contains several adjacent parcels. This result is a function of the tradeoff between risk and the benefits of conserving biologically rich parcels. In the Oregon example, the benefit of including three pairs of adjacent parcels, many with a large number of species or the presence of rare species, outweighs the risk of both parcels burning in a single fire. For several other areas of this landscape, however, the spatially-correlated risk leads to locating reserve parcels at a distance from each other. With large fires, pests, diseases, and invasive species increasingly threatening species conservation in the western U.S., this analysis focuses attention on incorporating spatially-correlated risk from natural hazards in reserve design decisions that permit tradeoffs rather than imposing spatial constraints.

In probabilistic maximum coverage models, as here, the typical objective function maximizes the expected number of species in the reserve but conservation actors may consider other secondary objectives [43]. For example, a manager choosing amongst reserve networks that generate identical expected species conservation could choose the network that maximizes the chance that all species survive. In contrast, if a manager’s secondary goal is to minimize the chance that no species survive, the optimal reserve network contains no adjacencies in the spatially-correlated risk case. The results here demonstrate that the pattern of parcels in the optimal reserve varies with the additional goals of minimizing or maximizing the chance of extreme events and with the distribution of species across space.

To identify the tradeoffs across the possible secondary criteria, this framework could be nested within a multi-criteria decision analysis (MCDA) (see [44] and [45] for review articles).to mediate conflicts between goals (e.g., [46]). In our case, instead of solving for the highest expected number of species and then considering the impact on other criteria such as the probability of no species surviving, weights could be attributed to both of these objectives, and, by varying those weights, the analysis determines how the reserve network varies with different goals. Although standard RSS models do not permit consideration of tradeoffs among criteria, multi-objective programming [47] allows an RSS model to have two (or more) objectives. Assigning weights to these objectives, the analyst can choose among an optimal set of non-dominated alternatives, or Pareto-optimal solutions, based on the relative importance given to each objective [38].

In conclusion, spatially-correlated risk creates incentives to locate reserve sites at a distance from each other but the species distribution and objective function determine whether that incentive dominates the reserve pattern, even in the absence of benefits to connectivity. The distribution of species across space interacts with the spatial risk scenario to determine the optimal reserve network’s configuration. The popular prioritizing of hotspots for conservation, for example, can endanger total species conservation when hazards threaten such agglomerated species, as in some spatially-correlated risk cases. Although the objective of maximizing expected species does not always lead to differences in network structure between spatially-correlated and spatially-independent risk scenarios, adding other goals creates or increases that difference. If the goal includes minimizing the probability that no species will survive, locating reserve sites at a distance proves best for the spatially-correlated risk case because a single large, spreading disturbance cannot destroy habitat and species in two distant sites. If the goal includes maximizing the probability that all species survive, however, locating reserve sites near each other proves best in the spatially-correlated risk case because the probability of a large disturbance in any particular location is relatively small. The current reserve site selection literature’s emphasis on maximizing the expected number of species conserved may not adequately address concerns and goals of some conservation managers in the face of risk. With spatially-correlated risk settings becoming increasingly common, reserve network design decisions should reflect the interaction of that pattern of risk with the distribution of species and the manager’s objectives.

## Supporting Information

S1 Dataset Oregon presence/absence matrix data. (ZIP)
